# Functional assay for assessment of pathogenicity of *BAP1* variants

**DOI:** 10.1093/hmg/ddad193

**Published:** 2023-11-13

**Authors:** Pauliina E Repo, Michael P Backlund, Tero T Kivelä, Joni A Turunen

**Affiliations:** Eye Genetics Group, Folkhälsan Research Center, Biomedicum Helsinki, Haartmaninkatu 8, FI-00290, Helsinki, Finland; Ocular Oncology Service, Department of Ophthalmology, University of Helsinki and Helsinki University Hospital, Haartmaninkatu 4 C, PL220, FI-00029 HUS, Helsinki, Finland; Eye Genetics Group, Folkhälsan Research Center, Biomedicum Helsinki, Haartmaninkatu 8, FI-00290, Helsinki, Finland; Ocular Oncology Service, Department of Ophthalmology, University of Helsinki and Helsinki University Hospital, Haartmaninkatu 4 C, PL220, FI-00029 HUS, Helsinki, Finland; Eye Genetics Group, Folkhälsan Research Center, Biomedicum Helsinki, Haartmaninkatu 8, FI-00290, Helsinki, Finland; Ophthalmic Genetics and Rare Eye Diseases Service, Department of Ophthalmology, University of Helsinki and Helsinki University Hospital, Haartmaninkatu 4 C, PL220, FI-00029 HUS, Helsinki, Finland

**Keywords:** uveal melanoma, BAP1, BAP1-TPDS, HAP1, variant interpretation

## Abstract

**Background:**

Pathogenic germline variants in BRCA1-Associated Protein 1 (*BAP1*) cause BAP1 tumor predisposition syndrome (BAP1-TPDS). Carriers run especially a risk of uveal (UM) and cutaneous melanoma, malignant mesothelioma, and clear cell renal carcinoma. Approximately half of increasingly reported *BAP1* variants lack accurate classification. Correct interpretation of pathogenicity can improve prognosis of the patients through tumor screening with better understanding of BAP1-TPDS.

**Methods:**

We edited five rare *BAP1* variants with differing functional characteristics identified from patients with UM in HAP1 cells using CRISPR-Cas9 and assayed their effect on cell adhesion/spreading (at 4 h) and proliferation (at 48 h), measured as cell index (CI), using xCELLigence real-time analysis system.

**Results:**

In *BAP1* knockout HAP1 cultures, cell number was half of wild type (WT) cultures at 48 h (p = 0.00021), reaching confluence later, and CI was 78% reduced (p < 0.0001). BAP1-TPDS-associated null variants c.67+1G>T and c.1780_1781insT, and a likely pathogenic missense variant c.281A>G reduced adhesion (all p ≤ 0.015) and proliferation by 74%–83% (all p ≤ 0.032). Another likely pathogenic missense variant c.680G>A reduced both by at least 50% (all p ≤ 0.032), whereas cells edited with likely benign one c.1526C>T grew similarly to WT.

**Conclusions:**

BAP1 is essential for optimal fitness of HAP1 cells. Pathogenic and likely pathogenic *BAP1* variants reduced cell fitness, reflected in adhesion/spreading and proliferation properties. Further, moderate effects were quantifiable. Variant modelling in HAP1 with CRISPR-Cas9 enabled functional analysis of coding and non-coding region variants in an endogenous expression system.

## Introduction

Pathogenic germline variants in the BRCA1-Associated Protein 1 (*BAP1*) tumor suppressor gene cause a rare, life-threatening tumor predisposition syndrome (BAP1-TPDS, OMIM 614327) also known as BAP1 cancer syndrome as the majority of carriers develop malignancy [1, 2]. The carriers are mostly affected by at least one of the four most frequent BAP1-TPDS index tumors: malignant mesothelioma (MM, 27%), uveal melanoma (UM, 24%), cutaneous melanoma (CM, 17%) or clear cell renal cell carcinoma (RCC, 10%) [1]. The frequency of mostly benign BAP1-inactivated nevi (BIN) in patients with BAP1-TPDS may be as high as 75% [3]. Still, the clinical phenotype of the syndrome is evolving. By 2022, some 234 families and 175 pathogenic or likely pathogenic germline *BAP1* variants have been reported and the inclusion of other less frequent tumors in the BAP1-TPDS tumor phenotype is being discussed. Among these are squamous and basal cell carcinoma (7%), meningioma (2%), breast cancer, hepatic and cholangiocarcinoma, and ovarian cancer [1, 4–7]. Additionally, pathogenic *de novo* missense variants causing loss of function have been identified in association with the newly found and phenotypically variable syndromic neurodevelopmental disorder known as Kury-Isidor syndrome (OMIM 619762), with undetermined risk of cancer [8].

Correct interpretation of the pathogenicity of *BAP1* variants has four major advantages for society and those who carry one in the germline. First, it enables surveillance for early diagnosis to improve prognosis [1, 9, 10]. For example, UM likely metastasizes only after reaching a diameter of 3 mm [11] and larger tumors can worsen vision through secondary glaucoma [12, 13]. However, cancer screening programs are provided to patients with a pathogenic or likely pathogenic variant, leaving those with a variant of unknown significance (VUS) without surveillance. Second, screening of pathogenic variant carriers has likely economic benefits [9]. Third, specific treatments for tumors with BAP1 loss might emerge [14, 15]. Finally, correct interpretation of the pathogenicity of variants is essential for refining the BAP1-TPDS phenotype, because its tumor profile depends on observation of true pathogenic variant carriers.

Worldwide, the cohort of patients with BAP1-TPDS is still small and the variants mostly unique to each identified family rendering genotype-phenotype studies difficult. The American College of Medical Genetics and Genomics (ACMG)/Association for Molecular Pathology (AMP) guidelines are most widely used for interpretation of gene variants [16] in clinical and research laboratories. *BAP1* is intolerant to loss-of-function variants, and thus interpretation of null variants as pathogenic is straightforward [8]. Classification of missense, synonymous, and non-coding region variants ideally requires functional testing—with cDNA vectors, bacterial or overexpression systems—or samples from heterozygous variant carriers—not always available—in addition to segregation analysis. Between June, 2018, and May, 2023, the number of *BAP1* variants reported as a VUS in ClinVar has grown from 278 to over 1000 [17, 18]. Insufficient evidence to qualify a variant as pathogenic vs. benign has resulted in many being classified as VUS. Such variants do not inform clinical decisions, which complicates patient counseling and management. Further tools to avoid frequent classification as a VUS are needed.

Here we describe a mammalian cell -based tool to assist in interpretation and correct classification of *BAP1* variants. The CRISPR-Cas9 editing enables the analysis of both intronic and exonic *BAP1* variants in an endogenous expression system.

## Results

### *BAP1* is essential for optimal HAP1 cell fitness

**Figure 1:**
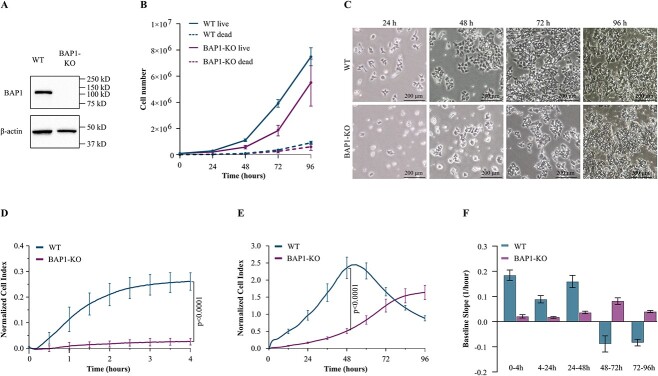
Proliferation and adhesion of BAP1 parental wild-type (WT) and knock-out (BAP1-KO) HAP1 cells (A) Western blot confirmed the loss of BAP1 in the BAP1-KO cells. (B) The growth of the WT and BAP1-KO cells was monitored for 4 days by counting their number every 24 h. The WT cells grew faster during the first 72 h before becoming compromised due to lack of nutrients and space (live cells, 24 h p = 0.0014, 48 h and 72 h p = 0.00021, 96 h p = 0.13, Mann-Whitney with Bonferroni correction). No significant difference was observed in the number of dead cells (p > 0.05). The graph represents the mean cell number ± SD at selected time points. (C) Phase contrast images taken every 24 h. (D) First 4 h normalized baseline cell index (CI) of the WT and BAP1-KO cells recorded by the xCELLigence real-time cell analysis (RTCA) system. Adhesion/spreading of the cells in the well caused an increase in the recorded impedance, reported as CI. The BAP-KO cell adhesion was reduced compared to the WT (4-h endpoint, p < 0.0001, Student’s *t*-test). The presented normalized baseline CI is the mean CI from two different runs using sample triplicates ± SD. (E) Cell index over 96 h shows the kinetic cell response curve of the WT and BAP1-KO cells. The initial increase in CI (0–4 h) indicates cell adhesion/spreading, followed by a brief lag phase before the cells start growing exponentially. CI of the WT cell at 48 h, just before reaching the peak, was 4.6 times the CI of the BAP1-KO cells (p < 0.0001, Student’s *t*-test). BAP1-KO cells reach confluence toward the end of day 4. Graph represents mean CI from two experiments done using sample triplicates ± SD. (F) Cell proliferation potential assessed by CI curve slope during the indicated time intervals. Slopes are from the xCELLigence RTCA software. WT cells window for maximal proliferation is between 24–48 h. Slopes were assessed from two runs conducted using sample triplicates ± SD.

To test how haploid HAP1 cells are affected by BAP1 loss, a cell line with a *BAP1* knockout and a WT control were acquired, and cell cultures were monitored for phenotypic differences. The knockout cell line BAP1-KO is homozygous for a 109 bp insertion in exon 5 of *BAP1* leading to loss of BAP1 expression (Fig. 1A). HAP1 proliferation was affected by this loss; 24 h after seeding, BAP1-KO cell quantity was ~30% less than that of the WT culture (Fig. 1B and C, p = 0.0014, Mann-Whitney with Bonferroni correction). At 48 and 72 h, the number of BAP1-KO cells was half of the WT cells (p = 0.00021). The difference in cell numbers was not significant anymore at 96 h once the cultures become deprived of nutrients and compromised (p = 0.13). Also, similar numbers of dead cells were observed at each measurement (24 h p > 0.99, 48 h p = 0.39, 72 h p = 0.40, 96 h p = 0.094). Thus, HAP1 cells with BAP1 knockout are viable but they reach confluence later than when BAP1 is intact.

We confirmed the growth rate difference between BAP1-KO and WT cells using the xCELLigence RTCA system, which measures well impedance as Cell Index (CI). Impedance is affected by adhesion, size, spreading, and proliferation of cells on the electrode-covered E-16 plate. Initial increase in the CI denoted the adhesion and spreading of the wild type HAP cells between 0–4 h (Fig. 1D) followed by a second peak signifying cell proliferation between ~4–48 h (Fig. 1E). BAP1 loss reduced HAP1 cell adhesion/spreading (Fig. 1D) and proliferation (Fig. 1E), considered here to be readouts of cell fitness. The CI of the BAP1-KO at was reduced 90% at 4 h and 78% at 48 h (4-h and 48-h endpoints, both p < 0.0001 Student’s *t*-test). The WT cells reached the maximum proliferation rate between 24–48 h and maximum density 48 h after plating, whereas BAP1-KO cells grew fastest at 48–72 h and reached a lower plateau during day 4 (Fig. 1E and F). In summary, BAP1 is essential for HAP1 cell optimal fitness.

### BAP1-TPDS-associated *BAP1* variants reduce HAP1 fitness

We validated HAP1 utility for diverse *BAP1* variant interpretation by measuring edited mutations c.67+1G>T, c.281A>G p.(H94R), c.680G>A p.(R227H), c.1526C>T p.(S509L), and c.1780_1781insT p.(G594Vfs^*^49) effect on BAP1 expression and HAP1 cell fitness. The five variants differ in their effect on BAP1 deubiquitination activity and nuclear localization, as we have previously identified through *in silico* and *in vitro* assays [19].

**Figure 2:**
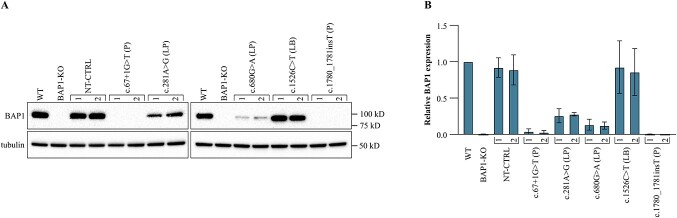
(A) Representative immunoblot of BAP1 protein expression in HAP1 cell lines edited to carry the five *BAP1* variants selected for the study. Each variant was assayed with two cell lines (1 and 2) originating from individual clones. (B) Bars represent the relative BAP1 protein expression quantified from three individual western blots (±SD). The NT-CTRL and cells with the likely benign c.1526C>T had ~10% reduction in the mean BAP1 protein expression which was considered within the normal range. The likely pathogenic variants c.281A>G and c.680G> A reduced expression ~73% and ~87%. The pathogenic variants c.67+1G>T and c.1780_1781insT reduced the BAP1 expression from barely detectable to non-existent. P, pathogenic; LP, likely pathogenic; LB, likely benign.

**Figure 3:**
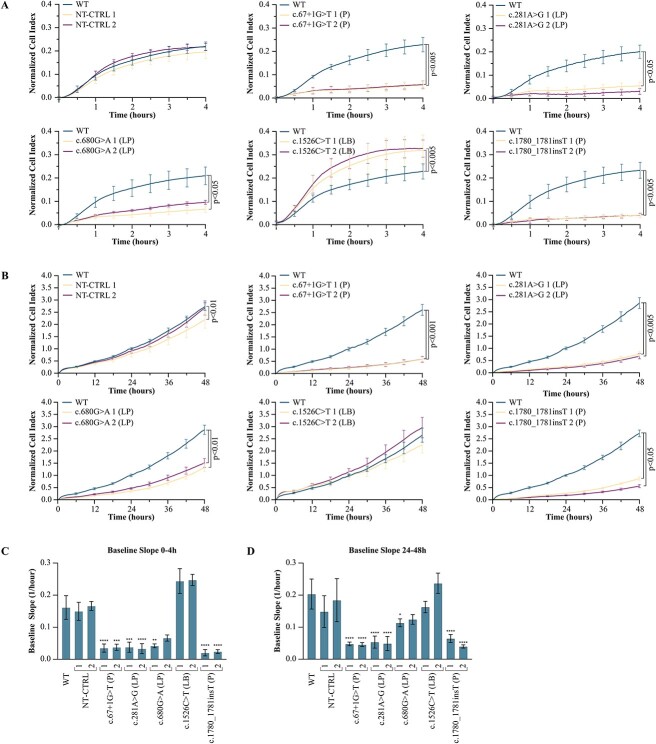
Normalized baseline cell index (CI) of the HAP1 cells edited to carry the five *BAP1* variants selected for the study, representing (A) the first 4 h and (B) 48 h. Each mutant was run together with the parental wild-type (WT) cell line within a single E-plate 16 for comparison. The CI is the mean of three assay times run using four technical replicates (±SD). Media were changed around 24 h after cell seeding. (A) All cultures with *BAP1* variants showed altered adhesion (4-h endpoint, c.67+1G>T 1 p = 0.00015, c.67+1G>T 2 p = 0.0011, 281A>G 1 p = 0.015, c.281A>G 2 p < 0.0001, c.680G>A 1 p < 0.0001, c.680G>A 2 p = 0.032, c.1780_1781insT 1 p = 0.0013, c.1780_1781insT 2 p = 0.00029, Kruskal-Wallis test with Dunn’s multiple comparisons test), however, only the likely benign variant (LB) c.1526C>T increased it (4-h endpoint, c.1526C>T 1 p = 0.0040, c.1526C>T 2 p = 0.00092). (B) Pathogenic (P) and likely pathogenic (LP) variants c.67+1G>T (P), c.281A>G (LP), c.680G>A (LP) and c.1780_1781insT (P) reduced the proliferation of both assayed cell lines from ~50% to ~80% (48-h endpoint, c.67+1G>T 1 p = 0.00028, c.67+1G>T 2 p = 0.00063, c.281A>G 1 p = 0.0040, c.281A>G 2 p < 0.0001, c.680G>A 1 p < 0.0001, c.680G>A 2 p = 0.0079, c.1780_1781insT 1 p = 0.032 c.1780_1781insT 2 p < 0.0001). Slopes of the baseline CI curves at (C) 0 to 4 h and (D) 24 to 48 h intervals were taken from the xCELLigence real-time cell analysis software. Slopes of both cell lines with c.67+1G>T, c.281A>G and c.1780_1781insT show reduced (C) adhesion/spreading (c.67+1G>T 1 p < 0.0001, c.67+1G>T 2 p = 0.00017, c.281A>G 1 p = 0.00019, c.281A>G 2 p < 0.0001, c.1780_1781insT 1 & 2 p < 0.0001) and (D) proliferation (c.67+1G>T 1 & 2, c.281A>G 1 & 2, and c.1780_1781insT 1 & 2 p < 0.0001) compared to the parental WT. Only the slope of culture 1 with c.680G>A differed from the WT reflecting a more moderate effect of the variant on (C) adhesion/spreading (c.680G>A 1 p = 0.0026, c.680G>A 2 p = 0.16) and (D) proliferation (c.680G>A 1 p = 0.045, c.680G>A 2 p = 0.16). Slopes of the non-targeting control (NT-CTRL) and the c.1526C>T were WT like (4 h, NT-CTRL 1&2 p > 0.99, c.1526C>T 1 p = 0.23, c.1526C>T 2 p = 0.14, 48 h, NT-CTRL1 p = 0.85, NT-CTRL 2 p > 0.99, c.1526C>T 1&2 p > 0.99). ^*^, p < 0.05; ^*^^*^, p < 0.005; ^*^^*^^*^, p < 0.0002; ^*^^*^^*^^*^, p < 0.0001.

The two pathogenic founder null variants c.67+1G>T and c.1780_1781insT caused in HAP1 cells effects similar to the knockout in BAP1-KO cell line. The cell lines showed little to no BAP1 expression (Fig. 2A and B, Supplementary Fig. S1). Also, the adhesion/spreading (0–4 h, Fig. 3A and C), and growth (48 h, Fig. 3B and D) of the cells was reduced to levels equivalent with those of the BAP1-KO (Fig. 1D and E). The mean CI at 4 h was reduced 76% and 83% by c.67+1G>T and c.1780_1781insT on average (c.67+1G>T 1 p = 0.00015, c.67+1G>T 2 p = 0.0011, c.1780_1781insT 1 p = 0.0013, c.1780_1781insT 2 p = 0.00029, Kruskal-Wallis test with Dunn’s multiple comparisons test). Likewise, proliferation of the c.67+1G>T and c.1780_1781insT edited HAP1 cells decreased, the 48 h mean CI being reduced by ~78% and ~74% (c.67+1G>T 1 p = 0.00028, c.67+1G>T 2 p = 0.00063, c.1780_1781insT 1 p = 0.032, c.1780_1781insT 2 p < 0.0001).

Likewise, the two likely pathogenic missense variants c.281A>G and c.680G>A negatively affected the growth of HAP1 cells (Fig. 3A–D). The c.281A>G harboring cells retained ~27% of the BAP1 expression (Fig. 2A and B), and its adhesion/spreading and growth were comparable to those of BAP1-KO and the null mutant carrying cells. The variant decreased the mean CI 79% at 4 h (c.281A>G 1 p = 0.015, c.281A>G 2 p < 0.0001, Kruskal-Wallis test with Dunn’s multiple comparisons test) and 75% at 48 h (c.281A>G 1 p = 0.0040, c.281A>G 2 p < 0.0001). The exon 9 variant c.680G>A reduced normal BAP1 expression to ~13% but affected growth only moderately because the mean CI at 4 h decreased by 61% (c.680G>A 1 p < 0.0001, c.680G>A 2 p = 0.032, Kruskal-Wallis test with Dunn’s multiple comparisons test) and at 48 h 51% (c.680G>A 1 p = 0.000029, c.680G>A 2 p = 0.0079). Its relatively subtle effect was more pronounced later, depicted in the 24–48 h slope of the CI graph of the cells which was double that of the HAP1 cells harboring the catalytically inactive c.281A>G variant (Fig. 3D). It should be noted that this variant produced a protein product of lower molecular weight observed once (Supplementary Fig. S1). Because the function and significance of this product is unknown, we excluded it from the western blot (WB) quantifications.

The NT-CTRL cultures and the cells with the likely benign c.1526C>T variant were similar to WT. The cells showed normal BAP1 expression (Fig. 2A and B). The adhesion of the NT-CTRL cultures did not differ from the WT (NT-CTRL 1 p = 0.11, NT-CTRL 2 p>0.99, Kruskal-Wallis test with Dunn’s multiple comparisons test), however, the c.1526C>T harboring cells showed 41% increase in 4-h CI compared to the WT (c.1526C>T 1 p = 0.0040, c.1526C>T 2 p = 0.00092, Fig. 3A and C). The proliferation depicted by 48 h CI did not differ from the WT (NT-CTRL 2 p>0.99, c.1526C>T 1 p = 0.081, c.1526C>T 2 p = 0.31) except for the other NT-CTRL culture (NT-CTRL 1 p = 0.0054, Fig. 3B). The CI slopes during adhesion/spreading at 0–4 h (NT-CTRL 1&2 p>0.99, c.1526C>T 1 p = 0.23, c.1526C>T 2 p = 0.14, Fig. 3C) and exponential growth at 24–48 h (NT-CTRL 1 p = 0.85, NT-CTRL 2 p>0.99, c.1526C>T 1&2 p > 0.99, Fig. 3D) did not differ from the WT, and the cultures were considered WT-like.

**Figure 4:**
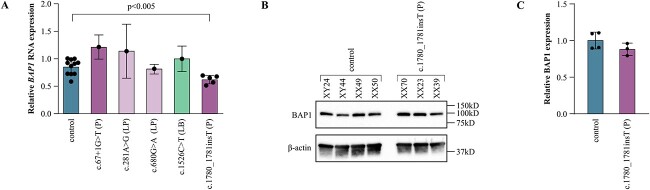
(A) Relative *BAP1* mRNA expression of controls and patient samples heterozygous for the five selected *BAP1* variants. RNA, extracted from blood, was quantified using reverse transcription PCR. Bars represent the mean BAP1 expression of the group (±SD), normalized to a control, and dots the mean each individuals independent runs. The c.1780_1781insT variant (n = 5) reduced *BAP1* mRNA expression moderately when compared to the controls (n = 11, p = 0.0032, Mann-Whitney U test). Differences of other variants were not tested (n = 1). (B) BAP1 protein expression in controls and c.1780_1781insT carriers. (C0 Quantified relative BAP1 protein expression from three western blots. The expression did not differ between controls and c.1780_1781insT carriers (p = 0.23). Bars represent the mean BAP1 expression of the group, relative to a control, and dots the mean expression of independent runs for each individual. P, pathogenic; LP, likely pathogenic; LB, likely benign.

Additionally, we undertook *in vitro* assays to examine the effect of the selected variants on BAP1 expression using samples from voluntary carriers. Only single carriers were available for RNA sampling regarding other variants than the c.1780_1781insT, and no further conclusions cannot be made from their effect (Fig. 4A). The blood samples from five c.1780_1781insT variant carriers had moderately reduced *BAP1* RNA expression (p = 0.0032, Mann-Whitney U test, Fig. 4A) while BAP1 protein expression was not altered in carrier fibroblast cultures (p = 0.23, Fig. 4B and C). Thus, we do not have enough evidence either to confirm or exclude that the variant may cause nonsense-mediated decay also *in vivo.*

## Discussion

Here, we provide lines of evidence of the utility of HAP1 cells as a model for improving the interpretation of *BAP1* variants, namely replication of variant-induced effects through quantifiable parameters of cell adhesion/spreading and proliferation characteristics observed in HAP1 cells. This *in vitro* assay provides supporting evidence to the ACMG/AMP guidelines used to guide clinical interpretation of *BAP1* variants in the context of dominant cancer predisposition and the neurodevelopmental Kury-Isidor syndrome. The assay results can be used as evidence to favor either a benign or pathogenic classification and can be considered especially relevant as regards non-truncating variants. Additionally, we consider HAP1-based variant modelling to be superior to the use of patient samples in which the WT allele can potentially mask the effect of the studied variant.

Of note, various methods are used for germline variant interpretation in other tumor predisposition syndromes. Data sharing and co-segregation analyses can be effective when multiple patients or entire families are available [20, 21]. Functional analyses have been developed to measure the effects of variants at the protein level; e.g. the effects of *BRCA1* variants on homology-directed repair [22], *TP53* variants on apoptosis [23], and *VHL* variants on cell and tumor xenograft growth [24]. Many such assays probe a single biological process and remain impractical or uneconomical for the interpretation of multiple germline variants. The development of CRISPR-Cas9 saturation genome editing allowed the interpretation of 4000 *BRCA1* [25] and 599 *BRCA2* [26] variants paving the way for high throughput functional testing of germline variants.

BAP1 is a deubiquitinase that regulates gene transcription, maintenance of genomic integrity and cell death [27–31]. In addition to large-scale proteome and transcriptome analysis, studies assaying BAP1 localization and deubiquitinating activity have uncovered several essential functions, diverse protein interactions, and downstream targets mediating BAP1 tumor suppression [28, 29, 31–34]. Here we aimed to develop a straightforward assay with one quantifiable variable that would favor variant interpretation as either benign or pathogenic, rendering the analysis of individual BAP1 functions and interactions unnecessary.

The first strength of our study is the discrimination of effects caused by a single nucleotide variant in a genomic context, enabled by CRISPR-Cas9 editing. Particularly, the assay informs interpretation of non-truncating single nucleotide variants. The observed differences in HAP1 fitness are the direct result of effects on BAP1 function or expression because the cells share the same genetic background, except for the edited variant. Second, the HAP1 assay can be upscaled for simultaneous analysis of multiple variants: such an approach has been undertaken using saturation genome editing as regards *BRCA1*, *BRCA2* and *NPC1* [25, 35, 36]. As 86% of the 1000 *BAP1* variants of uncertain significance reported in ClinVar single nucleotide missense alterations, the assay fulfils the need for an efficient tool to aid a major part of *BAP1* variant classification.

Moreover, we show that our method is suitable for detecting more subtle functional perturbations caused by missense variants with lesser effects. The BAP1 protein with the exon 9 missense variant p.(R227H) is expressed and likely is partially active, reflected by its lesser effect on HAP1 fitness. BAP1-TPDS is dominant with incomplete penetrance, and studies so far have failed to identify genotype-phenotype correlations [7]. Nevertheless, missense variant carriers as a rule develop fewer tumors, or a given type of tumor at a later age [17]. Identification of missense variants with effects more minor than those of null variants is likely to provide further insights into BAP1-TPDS tumor profile and prevalence. More variants should be assayed in detail to determine a possible threshold for pathogenicity. This also necessitates collection of more clinical family data worldwide.

Our study has some limitations most of which were taken into consideration in the experimental design. First, the assay reflects only effects in HAP1, and potential cell-type dependent effects on regulation and splicing, if any, cannot be observed. Second, cultured HAP1 cells revert to diploid, and the ploidy status of cells affects their fitness: diploid cells are larger, spread out more, and proliferate faster [37]. Also, contradictory data on the necessity of BAP1 for HAP1 cells exists [38] although our data agree with the initial identification of genes indispensable for HAP1 cells [39]. To minimize any bias caused by cultures with mixed ploidy, we passaged the edited cells to establish stable diploid genomes before performing our experiments. Third, the impedance measured by the xCELLigence is affected at least by cell size, flatness, adhesion, and proliferation. To differentiate individual effects, we measured cell adhesion strength before active proliferation, and the effect solely on proliferation was quantified using the BAP1-KO cell line (Fig. 1B). Lastly, CRISPR-Cas9 engineering of cell lines often mandates the inclusion of additional silent mutations to prevent recleavage of readily edited sites and to aid in correct clone selection [40]. Such included variants are synonymous, i.e. they presumably have no effect. Because our observations were supported by previously published evidence, we conducted no further experiments to estimate the possible effects of the silent mutations.

Findings not examined further herein include potential increase in adhesion/spreading of the HAP1 cells with exon 13 c.1526C>T p.(S509L). This variant does not impair BAP1 nuclear localization or enzymatic activity and is classified as likely benign (PM2, BP1, BS3). The serine-509 is phosphorylated [41] and located within a FOXK1/K2 interaction site [42]. The phosphorylation of BAP1 is important for its FOXK1/K2 interaction [42]. This interaction was not assayed and changes in it cannot be ruled out. Also, the effect of the selected variants on BAP1 deubiquitination activity and nuclear localization was not assayed because we had previously quantified them in our laboratory using other methods [19].

In summary, we present a cell-based functional tool to aid the clinical interpretation of *BAP1* variants. The main benefit of the assay is the ability to observe the effects of a single genetic variant in an endogenous expression system in a genomic context. The assay is also scalable for simultaneous analysis of multiple variants. The tool might be invaluable in providing assistance for the interpretation of BAP1 variants, particularly in challenging scenarios where the application of the ACMG/AMP guidelines otherwise proves challenging.

## Materials and Methods

### Ethics statement

The project was approved by the institutional review board of the Hospital Region of Helsinki and Uusimaa, and it followed the tenets of the Declaration of Helsinki. All donors gave a written informed consent upon sampling.

### RNA extraction and quantitative reverse transcription polymerase chain reaction (RT-qPCR)

Whole blood was collected in PAXgene Blood RNA Tube intended for *in vitro* diagnostic (IVD) testing, from which we isolated RNA using PAXgene Blood RNA Kit IVD (both from PreAnalytix, Hombrechtikon, Switzerland). Isolated RNA was treated with DNA-free DNA Removal Kit (Invitrogen, Thermo Fisher Scientific, Waltham, MA) and subjected for cDNA synthesis using iScript cDNA synthesis kit (Bio-Rad Laboratories, Hercules, CA). RNA and cDNA were used as a template in S15 PCR to exclude any remaining DNA contamination in the RNA samples. Gene expression assays for TBP, GAPDH and BAP1 (Hs00427620_m1, Hs99999905_m1 and Hs01109276_g1, Applied Biosystems, Thermo Fisher Scientific, Carlsbad, CA) were performed with the CXF96 Real-Time system (Bio-Rad Laboratories). We run assays twice using triplicate samples. Technical replicates with a cycle difference>0.3 were excluded from the analysis. If results from the two runs deviated in fold change > 0.3, we conducted a third run. Expression levels were normalized to the mean of TBP and GAPDH using the 2ΔΔCt method. Nine healthy individuals without family history of BAP1-TPDS and two healthy relatives of UM patients not carrying the *BAP1* variant provided specimens used as controls.

### Cultured fibroblasts

We established fibroblast cell lines from skin biopsies obtained from three carriers of the *BAP1* c.1780_1781insT pathogenic variant and from four healthy controls. They were put in Dulbecco’s Modified Eagle Medium (DMEM; Gibco, Waltham, MA, or EuroClone, Pero, Italy) supplemented with 20% fetal bovine serum (FBS; Biowest, Nuaillé, France), 1% penicillin–streptomycin (Gibco) and 1% GlutaMAX (Gibco). Samples were dissected and the fragments were treated with 3 ml of 1000 U/ml Type II collagenase (*Clostridium histolyticum,* Gibco) for 2 h at 37°C. After collagen inactivation with cold media, tissue fragments and detached cells were pelleted by 150 × g centrifugation for 10 min at 4°C. The pellet was resuspended and the cells were cultured in the same media until third passage, after which the FBS was reduced to 10%. The sex and age of the donor at the time of sampling is given in the name of each cell line: XX_70 was treated for UM, XX_23 and XX_39 were variant carriers without history of BAP1-TPDS, and controls XY_24, XY_44, XX_49 and XX_50 had no family history of any BAP1-TPDS cancer but they were not tested for germline *BAP1* variants. All cell lines tested negative with LookOut Mycoplasma PCR Detection Kit (Sigma-Aldrich, St. Louis, MO). All experiments were conducted on cell cultures below passage 8.

### Assayed patient derived *BAP1* variants

**Figure 5:**
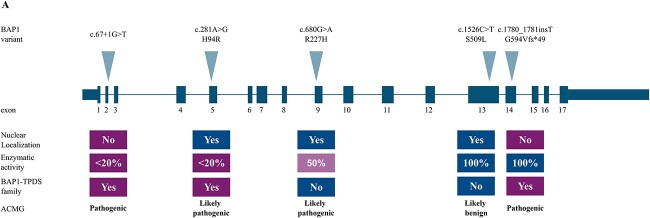
(A) Schematic representation of the five *BAP1* variants selected for the study. Information on their effect on BAP1 nuclear localization and enzymatic activity, occurrence in BAP1-TPDS families, and ACMG/AMP classification is shown below. Features supporting pathogenicity are emphasized in purple and the ones favoring benignity in blue.

We selected five *BAP1* variants, c.67+1G>T, c.281A>G p.(H94R), c.680G>A p.(R227H), c.1526C>T p.(S509L), and c.1780_1781insT p.(G594Vfs^*^49), to be assayed in the study. These previously published germline variants are identified from Finnish patients with UM and assessed *in silico* and *in vitro* for their effect on BAP1 deubiquitination activity and nuclear localization (Fig. 5) [19, 43].

Two likely Finnish founder variants, c.67+1G>T in intron 2 and c.1780_1781insT p.(G594Vfs^*^49) in exon 14, are classified as pathogenic (PVS1, PM2, PS3) according to the ACMG/AMP guidelines [16]. Both variants are found in several families with BAP1-TPDS and they cause loss of function (LOF) at the protein level by nearly eliminating BAP1 deubiquitinating activity, abolishing nuclear localization, or both (Fig. 5).

Two out of three selected missense variants, c.281A>G p.(H94R) (PM1, PP3, PM2, PS3) and c.680G>A p.(R227H) (PP3, PM2, BP1, PS3), are classified as likely pathogenic by the guidelines (Fig. 5). The exon 5 p.(H94R) variant, associated with BAP1-TPDS in a Finnish-Swedish family, is located in the large catalytically active ubiquitin carboxyl hydrolase (UCH) domain of BAP1 and nearly abolishes enzymatic activity [19]. Nevertheless, it is reported twice as VUS in ClinVar. The third, exon 9 p.(R227H) variant that locates close to the end of the UCH domain, reduces deubiquitinating activity by one half [19]. It is found in one family in which the index, affected by UM only, has no first- or second-degree relatives affected by BAP1-TPDS core cancers and no BAP1-TPDS is suspected. Updated pedigree is provided in Supplementary Fig. S2. The exon 13 missense c.1526C>T p.(S509L) variant is a likely benign (PM2, BP1, BS3) by the ACMG/AMP criteria. It does not affect BAP1 nuclear localization or enzymatic activity (Fig. 5) [19]. It is not associated with familial BAP1-TPDS. It is reported three times as VUS in ClinVar.

### HAP1 cells and CRISPR-Cas9 editing

HAP1 cells (RRID:CVCL_Y019) are adherent nearly haploid cells derived from a male patient with chronic myeloid leukemia [44, 45]. HAP1 cell line with *BAP1* knockout (BAP1-KO, HZGHC003319c004) and the parental wild type (WT) cell line were ordered from Horizon Discovery (Cambridge, UK). All HAP1 cells were cultured in Iscove’s Modified Dulbecco’s Medium (IMDM; Gibco) supplemented with 10% FBS (Biowest) and 1% penicillin-streptomycin (Gibco) and passaged before reaching 75% confluency. Cell lines tested negative for mycoplasma (Sigma-Aldrich).

We created HAP1 cell lines with the selected *BAP1* variants, and a wild-type control (NT-CTRL) using a non-human guide RNA (gRNA), by applying CRISPR-Cas9 technology in the parental WT cell line. The NT-CTRL was created to ensure that the editing and cloning protocol itself does not affect the growth properties of the cells. We used Alt-R CRISPR HDR Design Tool (Integrated DNA Technologies [IDT], Coralville, IA) to design the RNA and DNA oligos (Supplementary Table S1). HAP1 cells edited with c.281A>G, c.680G>A, and c.1526C>T required addition of silent mutations (Supplementary Table S1). The two-part gRNA complex was formed by heating 200 μM of Alt-R CRISPR-Cas9 tracrRNA-ATTO 550 with 200 μM Alt-R CRISPR-Cas9 crRNA for 5 min at 95°C, followed by hybridization at room temperature. The hybridized gRNA was incubated with an equal amount of Alt-R S.p. HiFi Cas9 Nuclease V3 for 15 min at room temperature to form a ribonucleoprotein (RNP) complex. The RNP complex with the Alt-R HDR Donor oligos (Supplementary Table S1) were electroporated with three 10 ms pulses of 1575 V (Neon Transfection System, Invitrogen) to parental WT HAP1 cells (2 000 000 cells/100 μl reaction). The transfected cells were seeded in antibiotic-free IMDM supplemented with 10% FBS and 30 μM Alt-R HDR Enhancer. The following day, ATTO 550 positive cells were sorted from the whole cell population and subjected to single cell cloning. DNA was isolated from the single cell clones (Blood DNA Isolation Kit, Geneaid Biotech, New Taipei City, Taiwan) and subjected to Sanger sequencing of *BAP1* coding regions [43] and the five most likely off-target areas, provided by IDT. We conducted experiments on two cell lines, originating from two individual colonies, homozygous for the desired alteration and negative for alterations in the analyzed off-target regions. More detailed protocol is available upon request form the corresponding author.

### Growth curve

We seeded 100 000 HAP1 WT and BAP1-KO cells on a 6-well plate in triplicates per each time point. Every 24 h, the cells were visualized by phase contrast imaging and harvested by trypsin for counting using Cellometer K2 Fluorescent Cell Counter (Nexcelom Bioscience, Lawrence, MA). The experiment was repeated with cells up to passage 19, and repeated three times.

### Adhesion and proliferation

We analyzed adhesion and proliferation of the HAP1 mutants and the parental WT using the xCELLigence Real-Time Cell Analysis (RTCA) DP system (Agilent Technologies, Santa Clara, CA). xCELLigence RTCA measure changes in well impedance caused by cell adhesion, spreading, size and number. Proliferating adherent HAP1 cells growth phases are recorded by impedance changes as cell index (CI) generating a curve distinctive to each cell line [46, 47]. Adequate cell number was optimized to detect adhesion/spreading, proliferation and cell death in CI curve during simultaneous runs for cells with normal and no BAP1 expression. We seeded 12 500 cells per well (100 μl) in electrode-covered E-plate 16 (Agilent) after measuring background impedance with media only. The plate was transferred to the RTCA plate station to record the impedance changes caused by initial cell adhesion and proliferation [47]. The obstructed electron flow, expressed as a cell index (CI), was monitored every two minutes for four hours, followed by one sweep per hour until 96 h. The growth medium was changed to a fresh one ~24 h after seeding. We compared WT and BAP1-KO by two independent assays with three technical replicates using cells at passage 13–15. We assayed each of the other mutants along with the parental WT cell line within a single plate to compare them by three independent assays. For this, four technical replicates with cells at passage 25 and above were used. Outliers were excluded from the analysis. CI curve slope values (at 0–4 h and 24–48 h) and baseline corrected CI values were exported from RTCA Software Pro (Agilent). Baseline CI values from runs were normalized to WT mean at read 143 (~24 h) after which the read for background was subtracted. Data from different runs were normalized using GraphPad Prism 9.4.1 (GraphPad Software, Boston, MA).

### Immunoblotting

For immunoblotting, we lysed cells in radioimmunoprecipitation assay (RIPA) buffer with protease inhibitors (cOmplete, Mini, EDTA-free Protease Inhibitor Cocktail, Roche Diagnostics, Risch-Rotkreuz, Switzerland). The cell debris was pelleted with 14 000 × g for 15 min at 4°C. Protein concentration of the lysates was quantified with Pierce BCA Protein Assay Kit (Thermo Fisher Scientific). Equal sample protein amounts were denatured and run in precast polyacrylamide gels (Bio-Rad, Hercules, CA). The proteins were transferred to polyvinylidene fluoride membranes (Biorad), followed by blocking in 5% milk or BSA in 0.1% Tris-buffered saline Tween-20 (TBST) for 1 h. Mouse monoclonal primary antibodies against the C-terminus of BAP1 (clone C-4; sc-28383, Santa Cruz Biotechnology, Dallas, TX, 1:500), beta-actin (clone 937215; MAB8929, R&D Systems, Bio-Techne, Minneapolis, MN, 1:50000), and rabbit polyclonal antibodies against alpha tubulin (ab4074, Abcam, Cambridge, UK, 1:5000) were incubated at 8°C overnight. After washes with 0.1% and 0.2% TBST, horseradish peroxidase-conjugated donkey anti-mouse or goat anti-rabbit IgG (HAF018 and HAF008, R&D Systems, 1:10000) or swine anti-rabbit (P0399, DAKO, Agilent Technologies, Santa Clara, CA, 1:8000) secondary antibodies and enhanced chemiluminescence substrate (SuperSignal West Femto Maximum Sensitivity Substrate, Thermo Fisher Scientific) were applied. More detailed protocol is available from the corresponding author upon request.

### Statistical analysis

Statistical tests were run using GraphPad Prism 9.4.1. All tests were 2-tailed, and *P* value < 0.05 was considered significant. Statistically significant difference in cell numbers for the growth curve were tested using Mann-Whitney U test followed by Bonferroni correction. WT and BAP1-KO endpoint CI values, and control and c.1780_1781insT BAP1 expression were compared using an Student’s *t*-test or Mann-Whitney U-test. xCELLigence endpoint CI values from the WT and the BAP1 mutant cultures were compared using the Kruskal-Wallis test with Dunn’s multiple comparisons test.

## Supplementary Material

SupplementaryFigureS1_ddad193

SupplementaryFigureS2_ddad193

SupplementaryTable_S1_ddad193

SupplementaryLegends_ddad193

## Data Availability

The data underlying this article will be shared on reasonable request to the corresponding author.
