# Supplementary figures and images for: Functional assay for assessment of pathogenicity of BAP1 variants
Source: Hum Mol Genet. 2023 Nov 13;33(5):426–34. doi: 10.1093/hmg/ddad193 (PMC10877462; doi:10.1093/hmg/ddad193)

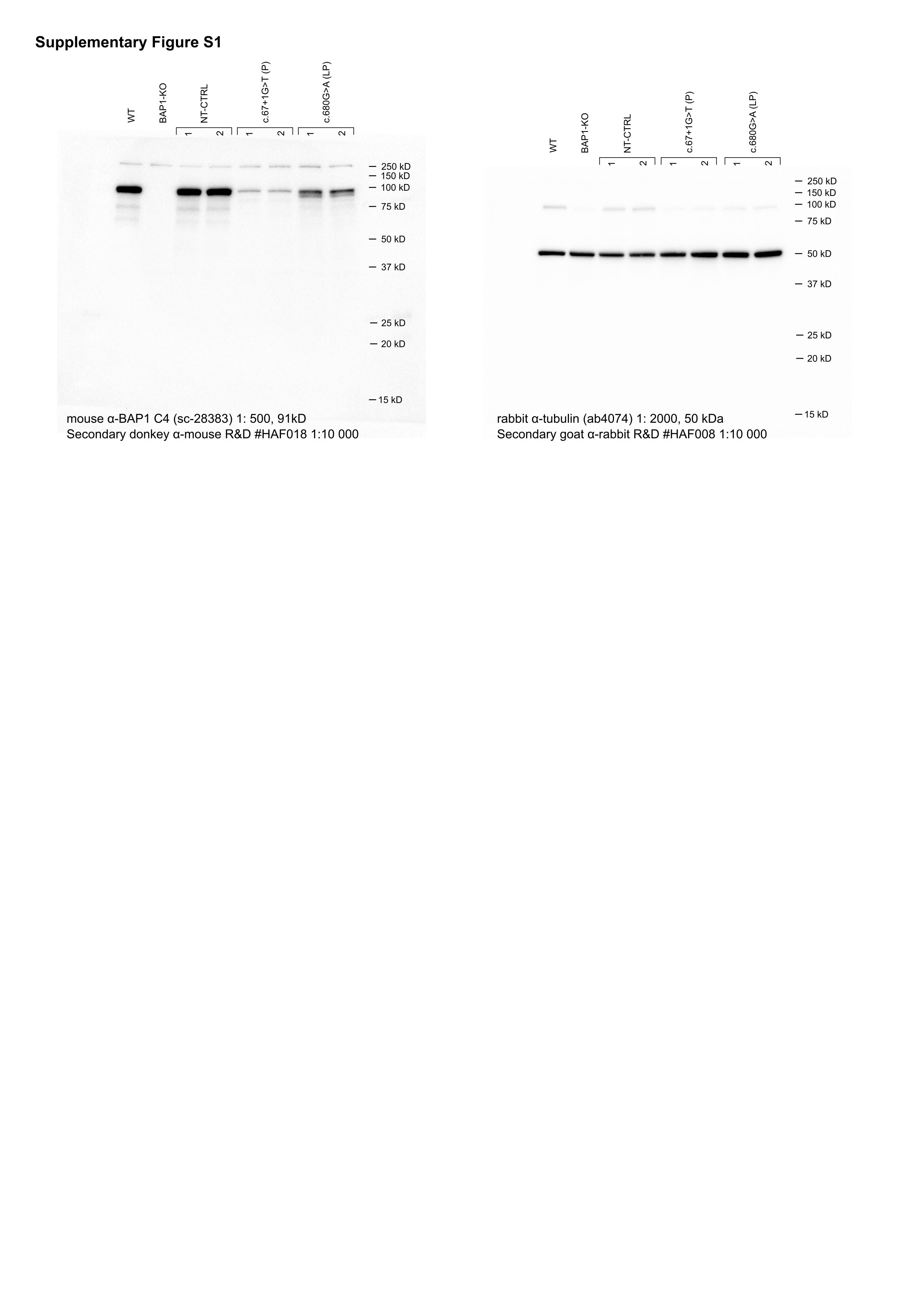

Supplement: SupplementaryFigureS1_ddad193 [file supplementaryfigures1_ddad193.zip › SupplementaryFigureS1_ddad193.png]
