# Supplementary material for: Functional assay for assessment of pathogenicity of BAP1 variants
Source: Hum Mol Genet. 2023 Nov 13;33(5):426–34. doi: 10.1093/hmg/ddad193 (PMC10877462; doi:10.1093/hmg/ddad193)
Supplement: SupplementaryLegends_ddad193 [file supplementarylegends_ddad193.doc]

**Supplementary Table S1** Detailed information on edited variants.

**Supplementary Figure S1** Immunoblot of HAP1 cell lines showing the BAP1 protein expression. Leakage of the pathogenic (P) splice site variant c.67+1G>T and the smaller protein product produced by HAP1 with likely pathogenic (LP) exon 9 c.680G>A is visible.

**Supplementary Figure S2** Updated pedigree of exon 9 c.680G>A (LP) variant carrier.
